# Influence of the Heterogeneity of the Core Material on the Local Instability of a Sandwich Panel

**DOI:** 10.3390/ma15196687

**Published:** 2022-09-27

**Authors:** Zbigniew Pozorski, Jolanta Pozorska

**Affiliations:** 1 Institute of Structural Analysis, Faculty of Civil and Transport Engineering, Poznan University of Technology, ul. Piotrowo 5, 60-965 Poznań, Poland; 2 Department of Mathematics, Faculty of Mechanical Engineering and Computer Science, Czestochowa University of Technology, Armii Krajowej 21, 42-201 Częstochowa, Poland

**Keywords:** sandwich panels, local instability, wrinkling, heterogeneous core, strain energy

## Abstract

The problem of local instability in the compressed facing of a sandwich panel is considered in the paper. The case of a facing resting on an infinite core is examined, but the validity of such a simplification has been discussed in detail. An energy approach is used to solve the problem. The general procedure for considering the influence of the core parameter variability on the value of stress causing the facing instability is presented. Expressions allowing us to calculate the wrinkling stress were derived, which was the main aim of the research. The heterogeneity of the core material is taken into account by using continuous functions describing the variability of the core material parameters. In the examples illustrating the theory, the exponential and polynomial functions were used. The examples are based on the actually measured elastic modules of the core. The presented considerations were extended to the analysis of strain energies, which confirmed that the properties of the layer adjacent to the facing (up to 2 cm thick) determine the value of the wrinkling stress. The paper presents an example of the optimization of core material parameters in which a change in the distribution of the core parameters led to an approximately threefold increase in the wrinkling strength of the sandwich panel.

## 1. Introduction

The subject of the analysis is sandwich panels made of thin but stiff facings and a thick but deformable core. The core can play various roles (e.g., insulation), but most of all it ensures the distance between the facings, which results in a significant increase in the stiffness and load-bearing capacity of the panels. These types of integrated structures are used in many industries. Although there are many mechanisms of failure within sandwich panels, in most cases the load-bearing capacity of the entire element is determined by local loss of stability, which has the form of wrinkling of the compressed facing. Compression in the facing can be caused by compression or bending of the sandwich element, but it can also be the result of thermal effects. In order to determine the appropriate load-bearing capacity of the panel, it is necessary to determine the stress that causes wrinkling of the facing.

The problem of determining the wrinkling stress in the case of a facing resting on a homogeneous core was solved by Hoff using the energy method and the linear decay function of the core deformation [1]. Plantema [2] used the same method but applied a different function, namely the exponential function. The differential equation method was applied by Allen [3]. In the case of an orthotropic core, the wrinkling stress for layered columns was determined in [4]. This solution was extended in [5], where orthotropic sandwich plates under general loading conditions were analyzed. A very similar problem of the wrinkling of composite-facing sandwich panels under biaxial loading was presented in [6]. Various core models were compared there, namely the Winkler elastic foundation, a linear decay model of Hoff–Mautner and an exponential decay model of Plantema. The multiaxial loading problem was also discussed in [7], but the case of the anisotropic core was considered. The strip model of the core was proposed in this work. The solution of the facing wrinkling problem for the sandwich panel with composite facings and orthotropic core was also presented in [8]. The authors derived the governing buckling equation using the energy method. In papers [9,10,11], the wrinkling problem was discussed using higher-order theories.

The influence of the heterogeneity of the core on the value of critical stresses was presented in [12]. Two cases were considered in this study: a piecewise functionally graded core and a continuously functionally graded core using the Hoff method. It was shown that a desirable increase of the wrinkling stress can be achieved using thin layers of a stiffer core adjacent to the facing. Interestingly, a similar conclusion was formulated in [13], which also used a layered core. The solution for four-point bending of a laminated beam with a physically non-linear core was presented in [14]. An energetic approach was applied, and the sinh function was used to describe the deformation of the core. The application of the extended high-order sandwich panel theory to the problem of the wrinkling of a sandwich panel with functionally graded core was presented in [15]. Two symmetrical types of functionally graded material distributions were considered in the paper.

Scientific research on wrinkling of thin facing is still of great interest because the problem of local loss of stability is important in many applications [16]. For example, many biological tissues are exposed to wrinkling. Therefore, the influence of material parameters on the formation of wrinkles is investigated [17]. A lot of work is devoted to designing controllable hierarchical wrinkling surfaces [18,19]. Improving anti-wrinkling properties is also important in the textile industry [20]. Of course, the value of the wrinkling stress also affects the solutions of sandwich elements with a hybrid core [21].

This paper considers the case of a facing resting on a core with variable material parameters. A continuous function of the variability of these parameters was assumed. The focus is on the exponential and polynomial functions, but there are no obstacles for it to be of any function. The main purpose of the research is to derive the equations for the wrinkling stress of a sandwich panel with the material properties of the panel core varying in thickness. In order to determine the critical stress, the energy approach was used similarly to [2]. The paper also presents an optimization problem consisting of determining the variability of material parameters, which ensures the maximum wrinkling stress under certain constraints. The optimization of material solutions seems to be very important in the context of modern applications of sandwich elements [22]. Any possible increase in the value of the critical stress practically results in a corresponding increase in the load-bearing capacity of the sandwich element.

This work can be considered in the context of previous analyses [23,24]. These articles did not deal a heterogeneous core but a homogeneous orthotropic core. The paper [23] presented a spatial numerical model of a sandwich panel for which an analysis of the influence of material parameters on the value of the wrinkling stress was performed. It has been shown that this stress depends on both the modulus of elasticity and the shear modulus of the core material. In [24], an analytical solution for a compressed facing resting on an orthotropic core was presented. Classical solutions to the problem with a homogeneous isotropic core were also compared, showing differences in relation to the strain energy of the core.

**Figure 1:**
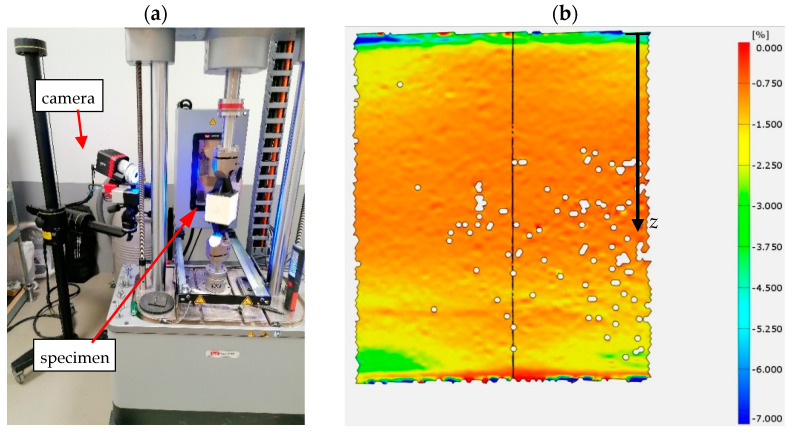
Measurement using the DIC method: (**a**) research stand and (**b**) exemplary results of longitudinal deformations.

**Figure 2:**
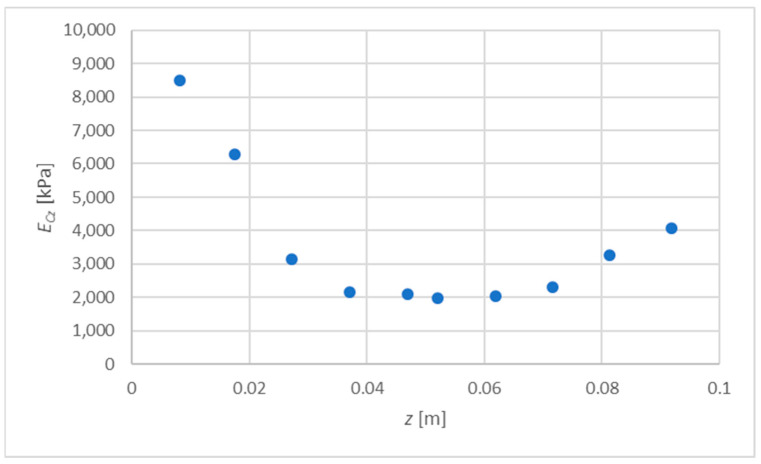
An example of the discrete distribution of the core elasticity modulus on the thickness of a sandwich panel; the *z*-axis is shown in Figure 1 and Figure 3.

The motivation to take up the presented issue was that the current technological possibilities of the production of sandwich panels allows for the production of a core with parameters varying in its thickness. The study of the variability of these parameters does not pose any major problems either. The digital image correlation (DIC) method is commonly used [25]. Figure 1 shows an example of the measurement results made with the ARAMIS 6M apparatus, which can later be analyzed using the GOM Correlate 2020 program. As a result, the values of the modulus of elasticity averaged for a given core layer are determined. The discrete values of material parameters are given for a specific point location on the core thickness. An exemplary distribution is shown in Figure 2.

One of the key problems that manufacturers of sandwich panels are currently working on is to obtain such a distribution of the core parameters that will ensure the maximum load-bearing capacity of the sandwich element with the minimum use of raw materials. Due to the growing technological possibilities of producers and a great emphasis on sustainable development of the industry, the issue of optimal distribution of the core material presented in the article seems to be strongly justified.

## 2. Formulation of the Problem

**Figure 3:**
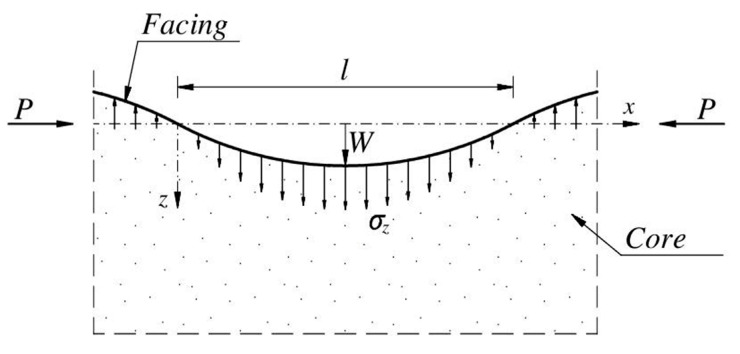
Assumed (sinusoidal) wrinkling of the facing resting on the infinite core.

During the bending of a sandwich panel with thin facings, one facing is compressed and the other is stretched. The case of unidirectional compression of the ideally flat facing is considered. The influence of initial irregularities of facing was no taken into account. A detailed analysis of this issue will be the subject of further research. Due to compression, the instability of the facing occurs, which has the character of periodic wrinkling. In order to determine the critical stress causing the local instability (wrinkling stress), the facing resting on the infinite core is examined (Figure 3). Such a simplification is allowed when the core is so thick that deformations of one facing do not affect the other facing. This issue will be discussed in an example.

Linear constitutive relationships were assumed for the facing and core materials. It was also assumed that the properties of the core change continuously along its thickness. In general, the modulus of elasticity $E_{Cz}$ (in the direction perpendicular to the facing) is independent of the shear modulus $G_{Cxz}$. As in [2,12]. Core deformations in the longitudinal direction (along the x-direction) were not included in the energy equilibrium equation. In other words, any vertical lines indicated before deformation remain vertical during wrinkling. The justification for this approach can be found, inter alia, in [24].

## 3. Determination of the Wrinkling Stress

### 3.1. General Equations

In order to determine the wrinkling stress, the energy approach was used in this paper. The basic Equations (1)–(11) can be found in [2].

Assume that the form of the facing deformation $w_{F}\left(x\right)$ is sinusoidal (W—deformation amplitude):

(1) $$w_{F}\left(x\right)=W\sin\frac{\pi x}{l},$$

and the core deformation $w_{C}\left(x,\;z\right)$ disappears exponentially (k>0):

(2) $$w_{C}\left(x,\;z\right)=w_{F}\left(x\right)e^{-kz}=We^{-kz}\sin\frac{\pi x}{l}.$$

The energy balance equation for the system shown in Figure 3 can be written (per unit width and over the length l) as:

(3) $$U_{F}+U_{C}=U_{P},$$

where:

(4) $$U_{F}=\frac{1}{2}\int_{0}^{l}B_{F}{\left(\frac{\partial^{2}w_{F}}{\partial x^{2}}\right)}^{2}dx$$

is the strain energy of the facing due to bending,

(5) $$U_{C}=\frac{1}{2}\int_{0}^{\infty }\int_{0}^{l}\frac{1}{E_{Cz}}\sigma_{Cz}^{2}dxdz+\frac{1}{2}\int_{0}^{\infty }\int_{0}^{l}\frac{1}{G_{Cxz}}\tau_{Cxz}^{2}dxdz$$

is the strain energy of the core, and

(6) $$U_{P}=\frac{1}{2}\int_{0}^{l}P{\left(\frac{\partial w_{F}}{\partial x}\right)}^{2}dx=\frac{PW^{2}\pi^{2}}{4l}=\sigma_{Fx}\frac{t_{F}W^{2}\pi^{2}}{4l}$$

is the work done by applied load P. Load P in (6) is expressed as the product of the normal stress in the facing $\sigma_{Fx}$ and the thickness of facing $t_{F}$. The symbol $B_{F}$ denotes the facing bending stiffness per unit width. For a beam it equals $E_{F}t_{F}^{3}/12$, for a plate it is $\frac{E_{F}t_{F}^{3}}{12\left(1-\nu_{F}^{2}\right)}=E_{F}^{*}t_{F}^{3}/12$, where $E_{F}$ is modulus of elasticity and $\nu_{F}$ is Poisson’s ratio of the facing material. The transverse normal stress $\sigma_{Cz}$ and the shear stress $\tau_{Cxz}$ in the core are:

(7) $$\sigma_{Cz}=E_{Cz}\frac{\partial w_{C}}{\partial z}=-E_{Cz}kWe^{-kz}\sin\frac{\pi x}{l},$$

(8) $$\tau_{Cxz}=G_{Cxz}\frac{\partial w_{C}}{\partial x}=G_{Cxz}\frac{\pi }{l}We^{-kz}\cos\frac{\pi x}{l},$$

where the modulus of elasticity $E_{Cz}$ (in the direction perpendicular to the facing) and shear modulus $G_{Cxz}$ (in plane *x*-*z*) of the core material are functions of the position variable *z*.

After substituting (4)–(8) to (3) and several transformations, we obtain the expression for the normal stress in the facing $\sigma_{Fx}$, which is a function of two variables, k and l:

(9) $$\sigma_{Fx}=\frac{E_{F}t_{F}^{2}\pi^{2}}{12l^{2}}+\frac{k^{2}l^{2}}{\pi^{2}t_{F}}\int_{0}^{\infty }E_{Cz}e^{-2kz}dz+\frac{1}{t_{F}}\int_{0}^{\infty }G_{Cxz}e^{-2kz}dz.$$

For any functions $E_{Cz}\left(z\right)$ and $G_{Cxz}\left(z\right)$, the wrinkling stress $\sigma_{w}$ is equal to $\sigma_{Fx}$ while meeting the conditions for the minimum:

(10) $$\frac{\partial \sigma_{Fx}}{\partial l}=0,$$

(11) $$\frac{\partial \sigma_{Fx}}{\partial k}=0.$$

From condition (11), $l^{2}$ can be determined, which is then substituted into (10). Finally, the optimal k and l are obtained, resulting in the wrinkling stress $\sigma_{w}=\sigma_{Fx}$. Below are presented solutions for two types of functions (exponential and polynomial) describing $E_{Cz}\left(z\right)$ and $G_{Cxz}\left(z\right)$.

### 3.2. Exponential Function of Material Parameters

Let the exponential form of the core parameters variability be assumed:

(12) $$E_{Cz}\left(z\right)=Ee^{-mz},$$

(13) $$G_{Cxz}\left(z\right)=Ge^{-nz},$$

where E, G, m and n are positive constants. Then Equation (9) takes the form:

(14) $$\sigma_{Fx}=\frac{E_{F}t_{F}^{2}\pi^{2}}{12l^{2}}+\frac{k^{2}l^{2}}{\pi^{2}t_{F}}\cdot \frac{E}{2k+m}+\frac{1}{t_{F}}\cdot \frac{G}{2k+n}$$

and optimality conditions (10) and (11) take the form:

(15) $$l^{2}=\frac{G}{E}\cdot \frac{{\left(2k+m\right)}^{2}}{{\left(2k+n\right)}^{2}}\cdot \frac{\pi^{2}}{k\left(k+m\right)},$$

(16) $$\frac{{\left(2k+n\right)}^{4}}{{\left(2k+m\right)}^{3}}\cdot {\left(k+m\right)}^{2}-\frac{12\cdot G^{2}}{E_{F}t_{F}^{3}E}=0.$$

To find the wrinkling stress, one should find k (16), then $l^{2}$ (15) and $\sigma_{w}=\sigma_{Fx}$ (14). 

For the special case m=n, the Equation (16) becomes the cubic equation with respect to k. This equation can be solved by the Cardano method. It can be shown that Equation (16) has one real solution:

(17) $$k=-\frac{5}{6}m+\sqrt[{3}]{\frac{m^{3}}{216}+\frac{A}{2}-\sqrt{\frac{A}{16}\left(\frac{m^{3}}{27}+A\right)}}+\sqrt[{3}]{\frac{m^{3}}{216}+\frac{A}{2}+\sqrt{\frac{A}{16}\left(\frac{m^{3}}{27}+A\right)}},$$

where constant A>0 is:

(18) $$A=\frac{12\cdot G^{2}}{E_{F}t_{F}^{3}E}.$$

### 3.3. Polynomial Function of Material Parameters

The general form of a polynomial describing the variability of the core parameters is taken as:

(19) $$E_{Cz}\left(z\right)=a_{1}z^{2}+b_{1}z+c_{1},$$

(20) $$G_{Cxz}\left(z\right)=a_{2}z^{2}+b_{2}z+c_{2},$$

where $a_{1}$, $b_{1}$, $c_{1}$, $a_{2}$, $b_{2}$ and $c_{2}$ are constants. Then Equation (9) takes the form:

(21) $$\sigma_{Fx}=\frac{E_{F}t_{F}^{2}\pi^{2}}{12l^{2}}+\frac{k^{2}l^{2}}{\pi^{2}t_{F}}\cdot \left(\frac{a_{1}}{4k^{3}}+\frac{b_{1}}{4k^{2}}+\frac{c_{1}}{2k}\right)+\frac{1}{t_{F}}\cdot \left(\frac{a_{2}}{4k^{3}}+\frac{b_{2}}{4k^{2}}+\frac{c_{2}}{2k}\right)$$

and optimality conditions (10) and (11) lead to:

(22) $$l^{2}=\pi^{2}\cdot \frac{\frac{3a_{2}}{4k^{4}}+\frac{b_{2}}{2k^{3}}+\frac{c_{2}}{2k^{2}}}{c_{1}-\frac{a_{1}}{k^{2}}},$$

(23) $$\left(\frac{a_{1}}{4k^{3}}+\frac{b_{1}}{4k^{2}}+\frac{c_{1}}{2k}\right)\cdot {\left(\frac{3a_{2}}{4k^{4}}+\frac{b_{2}}{2k^{3}}+\frac{c_{2}}{2k^{2}}\right)}^{2}-\frac{E_{F}t_{F}^{3}}{12k^{2}}\cdot {\left(c_{1}-\frac{a_{1}}{k^{2}}\right)}^{2}=0.$$

If k is find from Equation (23), $l^{2}$ (22) and $\sigma_{w}=\sigma_{Fx}$ (21) are also obtained.

## 4. Examples

### 4.1. Material Parameters

**Figure 4:**
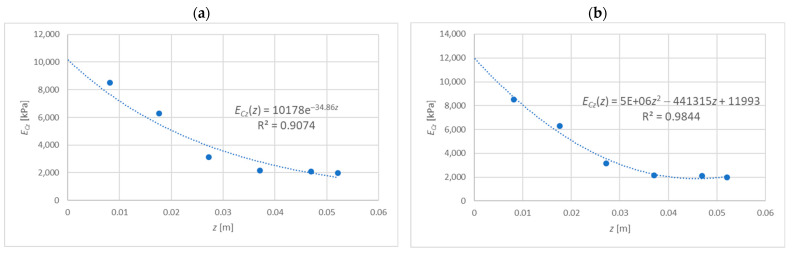
Function $E_{Cz}\left(z\right)$: (**a**) exponential, (**b**) polynomial of degree 2.

The distribution of the elasticity modulus presented in Figure 2 is considered, which will be described with the exponential function or the polynomial of degree 2. A certain disadvantage of the exponential function is that it correctly reflects the variability of material parameters only when the values disappear along with the variable z. For this reason, for the determination of $E_{Cz}\left(z\right)$ and $G_{Cxz}\left(z\right)$, only data for six points (up to a depth of z=0.0521 m) were taken into account; see Table 1, Figure 4.

For the sake of simplicity, it is assumed that the function $G_{Cxz}\left(z\right)=\frac{E_{Cz}\left(z\right)\;}{2\left(1+\nu_{C}\right)}$, where $\nu_{C}=0.3$. This also means that m=n in (12)–(16). Calculations were performed for the established $E_{F}=2.1\times 10^{-8}$ kPa and $t_{F}=0.0005$ m. These are typical parameters of manufactured sandwich panels.

### 4.2. The Exponential Function

From Figure 4a, the following function parameters can be read: E=10,178 kPa and m=34.86 [1/m], which means G=3915 kPa. For these parameters, one obtains: k=41.54 [1/m], l=0.03459 [m], $\sigma_{w}=138.6$ [MPa].

### 4.3. The Polynomial Function

**Figure 5:**
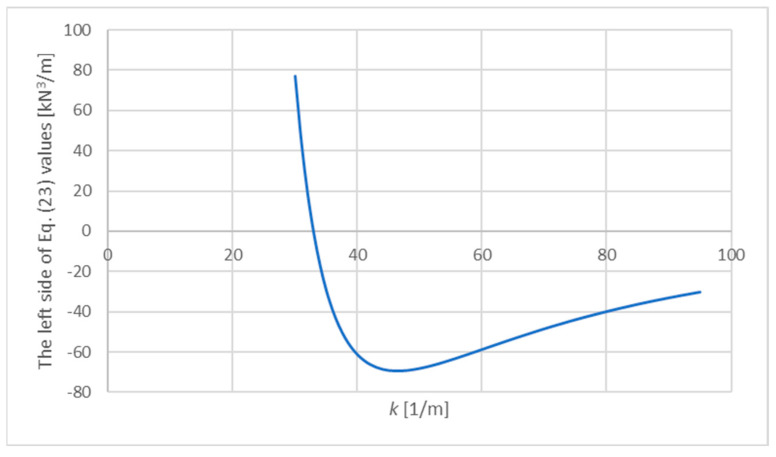
The values of the left side of Equation (23) presented as a function of the variable k.

From Figure 4b, the following function parameters can be read: $a_{1}=5,000,000$ kN/m^4^, $b_{1}=-441,315$ kN/m^3^, $c_{1}=11,993$ kN/m^2^, which means $a_{2}=1,923,077$ kN/m^4^, $b_{2}=-169,737$ kN/m^2^ and $c_{2}=4612.7$ kN/m^2^. For these parameters, a quantity k is sought which will satisfy the condition (23). The values of the left side of Equation (23) presented as a function of the variable k are shown in Figure 5.

It turns out that for k values lower than 30, the expression quickly goes to infinity, while for large values of k, expression (23) is negative and goes to zero. The condition (23) is satisfied for: k=32.94 [1/m], which means: l=0.03612 [m], $\sigma_{w}=154.9$ [MPa].

### 4.4. Comparison of the Results

It is worth noting now that the discrepancy between the results obtained for two different functions does not result from the determination method itself, but from differences in the values of the respective functions $E_{Cz}\left(z\right)$ and $G_{Cxz}\left(z\right)$. It is enough to note that exponential functions have values E(z=0)=10,178 kPa, G(z=0)=3915 kPa, while polynomial functions obtain values E(z=0)=11,993 kPa, G(z=0)=4612.7 kPa. The difference between the wrinkling stresses 138.6 MPa and 154.9 MPa is reflected very accurately (error of 0.1%) by the known expression $\sigma_{w}=\alpha \sqrt[{3}]{E_{F}EG}$, where α is a certain factor. On this basis, it is possible to confirm the findings of other authors [12,13], that the wrinkling stress is determined mainly by the modules for the core layer directly adjacent to the facing.

## 5. Energy Considerations

**Figure 6:**
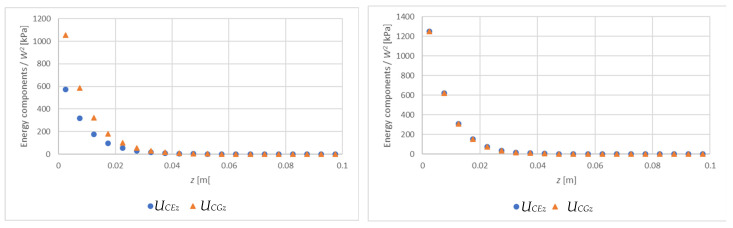
The components of $U_{C}$ (divided by $W^{2}$) determined for the successive layers of the core with a thickness of 0.005 m: (**a**) m=34.86 [1/m], (**b**) m=0 [1/m].

In order to trace the relationship between the energies expressed in (4) and (5), we consider two cases: the exponential function from the example above (E=10,178 kPa and m=34.86 [1/m]) and the constant function (E=10,178 kPa and m=0 [1/m]). For these two cases, Figure 6 shows the $U_{C}$ components (divided by $W^{2}$), determined for the successive layers of the core with a thickness of 0.005 m ($z_{2}-z_{1}=0.005$):

(24) $$U_{CEz}=\frac{1}{2W^{2}}\int_{z_{1}}^{z_{2}}\int_{0}^{l}\frac{1}{E_{Cz}}\sigma_{Cz}^{2}dxdz,$$

(25) $$U_{CGz}=\frac{1}{2W^{2}}\int_{z_{1}}^{z_{2}}\int_{0}^{l}\frac{1}{G_{Cxz}}\tau_{Cxz}^{2}dxdz.$$

**Figure 7:**
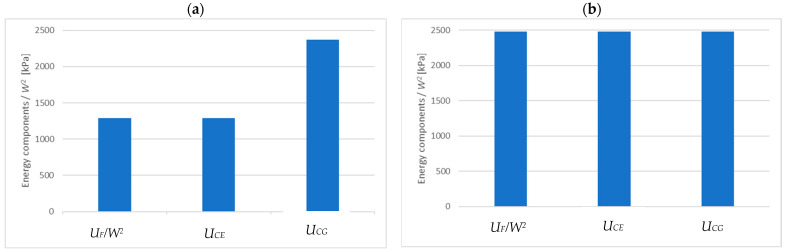
The comparison of the energies (divided by $W^{2}$) for the exponential distribution of $E_{Cz}\left(z\right)=10178\cdot e^{-mz}$: (**a**) m=34.86 [1/m], (**b**) m=0 [1/m].

**Figure 8:**
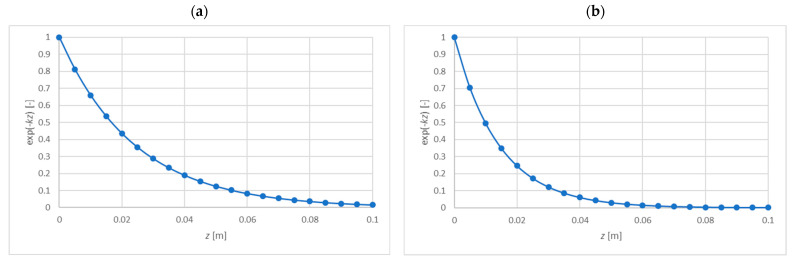
The comparison of the expression $e^{-kz}$ for the exponential distribution of $E_{Cz}\left(z\right)=10178\cdot e^{-mz}$: (**a**) m=34.86 [1/m], (**b**) m=0 [1/m].

Figure 7 presents the comparison of the energy $U_{F}/W^{2}$ with energies $U_{CEz}$ and $U_{CGz}$ summed over the entire thickness of the core and denoted as $U_{CE}$ and $U_{CG}$, respectively. Figure 8 compares the expression $e^{-kz}$, which illustrates the disappearance of displacements in the core.

On the basis of Figure 6, Figure 7 and Figure 8, it can be concluded that in both cases the vast majority of the core strain energy (over 90%) is stored in a 2 cm thick layer adjacent to the facing, which confirms the observations formulated in [12,13]. Core deformation decays more slowly than strain energy does. In the case of a core with constant stiffness (Figure 8b), the reduction of displacements by 90% occurs for z=0.035 m; for a core with variable stiffness, a similar reduction takes place for z=0.06 m. Therefore, in the two presented cases, disregarding the influence of the tensile facing is justified when the core thickness is 7 cm and 12 cm, respectively. These thicknesses correspond to twice the depth of the zone of significant core deformation. Obviously, in the case of a constant stiffness core, both energy and displacement decay faster. It should also be noted that in the case of a core with constant material parameters $U_{F}=U_{CE}=U_{CG}$, while for a core with variable stiffness (decreasing with thickness) $U_{F}=U_{CE}$, but the energy $U_{CG}$ is higher than the others ($U_{CE}=U_{CG}$ only for k=0). This means that in the case of a core with decreasing stiffness, the wrinkling stress is slightly more determined by the shear strain energy $U_{CG}$ than the normal strain energy $U_{CE}$.

## 6. Optimal Distribution of Material Parameters

A core in which the parameters $E_{Cz}\left(z\right)$ and $G_{Cxz}\left(z\right)$ change exponentially (12) and (13) is analyzed. It is obvious that the stiffer the core, the higher the wrinkling stress value. However, let us introduce the following constraints (C, D—constants):

(26) $$\int_{0}^{\infty }E_{Cz}dz=\int_{0}^{\infty }Ee^{-mz}dz=\frac{1}{m}E=C,$$

(27) $$\int_{0}^{\infty }G_{Cxz}dz=\int_{0}^{\infty }Ge^{-mz}dz=\frac{1}{n}G=D.$$

which can be interpreted as the limit of the stiffness summed over the thickness of the core. To simplify the presentation, it can be assumed that m=n. Using (26), (27) and (15), it is possible to express the normal stress $\sigma_{Fx}$ as a function of the variable m (k also depends on m):

(28) $$\sigma_{Fx}=\frac{E_{F}t_{F}^{2}}{12}\cdot \frac{C}{D}\cdot k\left(k+m\right)+\frac{D}{t_{F}}\cdot \frac{m}{\left(k+m\right)}.$$

Bearing in mind that k is expressed by (17), the condition for obtaining the maximum stresses is:

(29) $$\frac{\partial \sigma_{Fx}}{\partial m}=\mathrm{0.}.$$

For the assumed values C=300 kN/m and D=300/2.6=115.385 kN/m, according to (28), the maximum wrinkling stress is obtained for m=142.4 and is $\sigma_{w}=230.77$ MPa.

**Figure 9:**
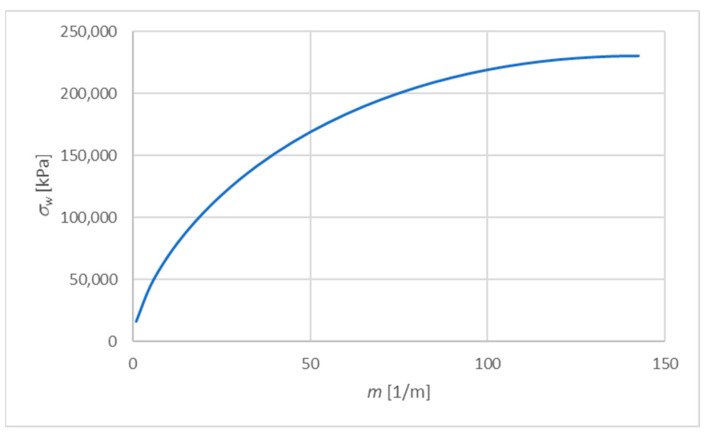
The dependence of $\sigma_{w}$ on m.

For comparison, for *m* = 10, which corresponds to the distribution of material parameters close to uniform, the wrinkling stress is 68.82 MPa. The dependence of $\sigma_{w}$ on m is shown in Figure 9.

**Figure 10:**
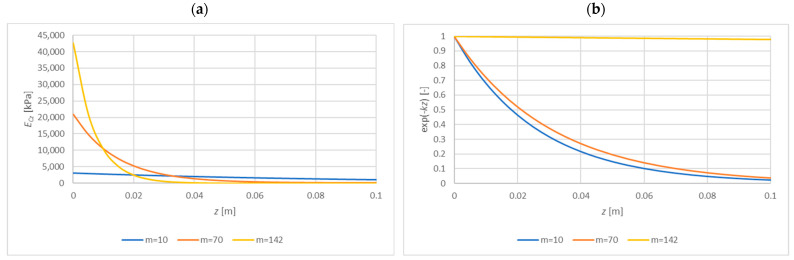
The influence of the parameter m on (**a**) $E_{Cz}\left(z\right)$, (**b**) $e^{-kz}$.

Interestingly, for the optimal m (in the sense (26)–(29)), the value of k is close to zero, and l increases significantly (it is on the order of a few meters—depending on the accuracy of m determination). Moreover, for optimal m, as z increases, the strain energy in the core disappears, but the core deformations ($e^{-kz}$) do not disappear at all but remain at the same level. A comparison of the distribution of $E_{Cz}\left(z\right)$ and $e^{-kz}$ for three different levels of m is presented in Figure 10. It seems as if the analyzed layered structure was trying to convey its deformations into the core. In practice, a second facing will appear at some distance from the facing to be compressed, disturbing these deformations.

## 7. Conclusions

The problem of the local instability (wrinkling) of the facing resting on the deformable core was analyzed in the paper. The case of uniaxial compression of the facing was considered. The core was assumed to be infinitely thick. Such a simplification, although it may turn out to be very limiting, is in many cases acceptable due to the rapid decay of the strain energy and deformation of the core.

In the first part of the paper, using the energy approach, the procedure for determining the wrinkling stress was presented in the case of a core with any variation in its material parameters. This procedure was used to obtain a solution for a core, in which material parameters are expressed with an exponential or polynomial function. The equations are illustrated with examples that use discrete values of core modules determined on its thickness. The presented procedure is general and can be applied to any function describing the variability of the core properties.

The considerations concerning the strain energy in the system showed the following:

- More than 90% of the strain energy of the core is stored to a depth of about 2 cm from the facing, which means that the value of the wrinkling stress is mainly determined by the layer adjacent to the facing;
- Core deformation is reduced by 90% at a depth of 6 cm (core with variable parameters) or at a depth of 3.5 cm (core with constant parameters), which means that considering the theoretical case of an infinitely thick core is acceptable in most cases;
- The strain energy of the facing deformation is always equal to the normal strain energy of the core; the shear strain energy of the core is equal to them only when the core parameters are constant across the thickness;
- In the case of non-linear (disappearing with thickness) distribution of core material parameters, the shear strain energy of the core has a decisive influence on the wrinkling stress.

The last part of the work presents an example of optimizing the distribution of core material parameters, the aim of which was to maximize the wrinkling stress. An optimal solution was obtained for the assumed class of exponential functions and for the introduced constraints. The solution is characterized by high material modules in the layers adjacent to the facing and a quick reduction of these modules in the thickness of the core.

The presented considerations explain the enormous and practical influence of the distribution of core material parameters on the load-bearing capacity of a complete sandwich panel. The derived equations can be directly used in the production of sandwich panels with a better distribution of the core material parameters.

## Figures and Tables

**Table 1:** Laboratory determined values of $E_{Cz}$ for the position z.

z **[m]**	0.00814	0.0176	0.0272	0.0371	0.0470	0.0521
$E_{Cz}$ **[kPa]**	8509	6282	3146	2173	2093	1994

## Data Availability

The data presented in this study are available on request from the corresponding author.
